# Research on parking sharing strategies considering user overtime parking

**DOI:** 10.1371/journal.pone.0233772

**Published:** 2020-06-10

**Authors:** Xin Huang, Xueqin Long, Jianjun Wang, Lan He

**Affiliations:** 1 College of Transportation Engineering, Key Laboratory of Transport Industry of Management Control and Cycle Repair Technology for Traffic Network Facilities in Ecological Security Barrier Area, Chang’ an University, Xi'an, Shan Xi, China; 2 Hefei Research Center of Urban and Rural Construction and Development, Hefei, China; Zhejiang University, CHINA

## Abstract

A parking sharing strategy is proposed to solve the problems of parking difficulty caused by the imbalance between parking spaces and parking demand. The vacant parking spaces of residential area can be efficiently utilized to meet the parking demands of those who are working at nearby or come for other activities based on the parking sharing strategy. The paper analyzes the distribution of vehicle arrival numbers and parking durations, then establishes a shared parking allocation model aiming to maximize the parking benefit considering the overtime-parking behavior of the parking users. Simulation methods are used to the analyze the relationship among the parking benefit, proportion of reserved parking, numbers of parking demand, acceptance rate of parking demand and utilization of shared parking spaces. Then, based on the principle of maximum parking benefit, we can determine the optimal proportion of reserved parking, number of shared parking spaces that should be purchased from the residents. Taking the utilization of shared parking spaces as an indicator, the validity of the static allocation principle is proved to be effective. Some allocation rules for parking demand are proposed to guarantees the maximum parking revenue and minimum impact on residents simultaneously.

## Introduction

The imbalance between parking demand and supply has increased gradually because of the rapid increase of motor vehicles, then parking difficulty become the critical problem that should be solved during the process of urban development. However, because of the restrictions of land, funds and other resources, parking demand cannot be completely satisfied by building parking facilities alone. Thus, parking sharing has provided a new idea to balance the parking supply and demand through staggering using the parking plots.

Parking sharing refers to the organizational form that parking spaces are utilized at different parking periods among different types of adjacent lands [1], which can balance the utilization of parking resources over time and space. Parking sharing study started in the 1980s. Howard [2] and Frank [3] discussed the feasibility of using parking facilities of adjacent construction when the parking demand is oversaturation during a certain period of time.

To improve the utilization of existing parking resources, parking sharing has gradually been regarded as a key traffic management strategy at home and abroad [4,5]. Taking residential area as an example, the parking behavior represents the obvious characteristic of going out in the morning and returning in the evening; therefore, the residential parking spaces are mostly idle during the working daytime. If the parking management company purchases the idle time of the private parking spaces and sales them to the public, utilization of parking resources can be greatly improved, the owners of parking plots and operators can both obtain certain benefits. Different types of parking facilities have differentiated sharing potentiality, that the parking facilities of offices, schools, restaurants and shopping malls have greater parking sharing potentiality [6].

For parking sharing, parking time window constraints are also the most critical problems that must be solved. A method of parking time window determination has been proposed after analyzing the time-varying characteristics and user’s parking selection for single land use type [7–10].

Some other methods have also been proposed to optimize the parking sharing system. Wang [11] established a sharing parking allocation method aiming to achieve the time-space balance, which combines land resources and uses objective investigation data. Zhu [12] proposed the parking allocation strategy using three-level Stackelberg game model, which aimed to obtain the maximum profit for the private owners and the minimum social cost for the government. Duan [13] proposed a Bilevel Programming model to achieve the coordinated control of parking spaces. The up level of the model aims at minimizing the difference of parking plots’ idle index at peak hour, and the low level aims at minimizing the average working distance after drivers’ parking. Zou [14] proposed a parking spaces allocation method considering both static and dynamic mechanisms.

Additionally, a large number of researchers have studied the parking sharing from different aspects. Some studies aimed at the maximum parking sharing benefits for the manager. Guo [15] analyzed the parking benefits considering the prospective loss of users who are refused to park, from the operators’ perspective of maximum parking profit and utilization of parking spaces. Similarly, Shao [16] established a parking benefits maximization model considering the parking purchases cost and the users’ compensation who return early, and optimized the amount of parking spaces purchased and sharing time through simulation. Li [17] studied the parking sharing mode aiming to obtain the greatest benefit under the condition of avoiding parking conflicts, and adjusted the sharing termination time and the shared parking opening rate according to the parking demand.

Utilization of parking plots is another objective that should be considered for parking sharing. Yao [18] established a parking space utilization optimization model, which is proved to be valid comparing with the method of first-come-first-served. Aiming at the minimum comprehensive occupancy rate of the parking plots in the combined land, Chen [19,20] established the space-time matching optimization model for parking supply and demand based on User Equilibrium Theory and then proposed the two-layer shared parking allocation model. Simply, Duan [21] constructed a allocation model aiming to maximize the sharing-capacity during peaking hours under the constraint of idle time.

Various parking modes will lead to different profits and utilization of parking sharing. Then, Duan [22] studied the parking choice game behavior of residential areas under the condition of limited resources, and constructed a coordinal control service model of shared parking. Zheng [23] considered the parking permits and floating pricing to adjust the dynamic parking demands, which can precisely regulate the occupancy rates of parking spaces. Yang [24] studied the parking capacity under two parking modes including reserved parking spaces and first-come-first-competition public parking spaces. Zhen [25] proved that parking charge and reservation can remarkably improve the comprehensive benefit using agent simulation.

Parking plots reservation and walking distances must be considered for parking sharing. Liu [26] studied the effect of parking spaces reservation on relieving congestion and reducing commuter parking time. Ran [27] studied the feasibility of parking sharing based on walking distance after parking.

Parking sharing ultimately needs to be realized through a smart parking system. Germán Martín [28] presented a smart parking system using occupancy simulator. And it is valuable and meaningful to the parking sharing platform.

At present, research of parking sharing focuses mostly on the operator's income, parking allocation optimization on the basis that all users strictly abide by the time reserved and parking demand is known in advance. However, some users cannot fully comply with the reserved parking periods because of unexpected factors, overtime parking may occur randomly. Then, the operator should set a certain number of reserved parking spaces to avoid the conflict of overtime parking. Additionally, how to selectively accept some parking demand is the principal issue that should be considered by the operator.

The article takes the combination land of residential area adjacent to commercial area as subjective, and a real-time sharing parking spaces allocation model aiming to maximize the operator’s benefits has been established, which considering external user's overtime parking behavior. Reserved shared parking spaces is introduced for avoiding the parking conflict caused by overtime parking. Numerical simulation has been adopted to analyze the relationships among the parking revenue, parking duration, parking demand and utilization of parking spaces. According to the analysis results, proportion of reserved shared parking spaces, number of purchased shared parking spaces and the amount of parking demand can be obtained from the perspective of operator’ maximum benefit. The model can provide the optimal allocation program to both ensure the maximum revenue for the operator and the minimum impact for the residents.

The remaining text is organized as follows. Section 1 gives a brief introduction to the parking characteristics, including the vehicle arrival numbers and parking durations. Next, we describe the shared parking problem, analyze the parking supply and demand in section 2. Allocation principles and process are proposed based on the balance theory of supply and demand in section 3. The allocation model aiming at benefit maximization of shared parking is established in section 4. Section 5 analyze the simulation results of shared parking. Section 6 concludes this paper and suggests some future work.

## Parking characteristics analysis

We selected an underground parking of Joy City shopping mall in Xi'an, Shan Xi Province, China for investigation. Joy City shopping mall is located at 777 Ci'en Road, Yanta District, Xi'an (west side of South Square of Great Wild Goose Pagoda). The parking data during a week of May 21 to 25, 2019 is obtained from the manager of the underground parking place. The parking data includes arrival time, leaving time and vehicle state (arrival or leaving) for every parking vehicle. No individual privacy is contained in the parking data. The vehicle arrival time and parking duration are matched by the license plate number, parking data of 8 hours from 8:00 a.m. to 18:00 p.m. during the five weekdays are used for deep analysis.

To understand the relationship of the vehicle arrival time and parking duration, correlation test between the two characteristic variables is conducted. The correlation coefficient can be expressed as:

$$\rho (t,\Delta t)=\frac{\sum_{i=1}^{m}(t_{i}-\bar{t})(\Delta t-\Delta \bar{t})}{\sqrt{\sum_{i=1}^{m}{\left(t_{i}-\bar{t}\right)}^{2}\sum_{i=1}^{m}(\Delta t-\Delta \bar{t}{)}^{2}}}$$ (1)

Where *t*_*i*_ and Δ*t* represent the arrival time and parking duration of the i^th^ user respectively. $\bar{t}$ and $\Delta \bar{t}$ are the corresponding average values of the arrival time and parking duration.

According to the investigation of working days, the correlation coefficient between the arrival time and the parking duration is between 0.13 and 0.28, which is less than 0.3. We can conclude that, the vehicle arrival time and parking duration are approximately statistically independent. Then, separate distribution models can be used to characterize the two random variables.

### Vehicle arrival analysis

**Fig 1:**
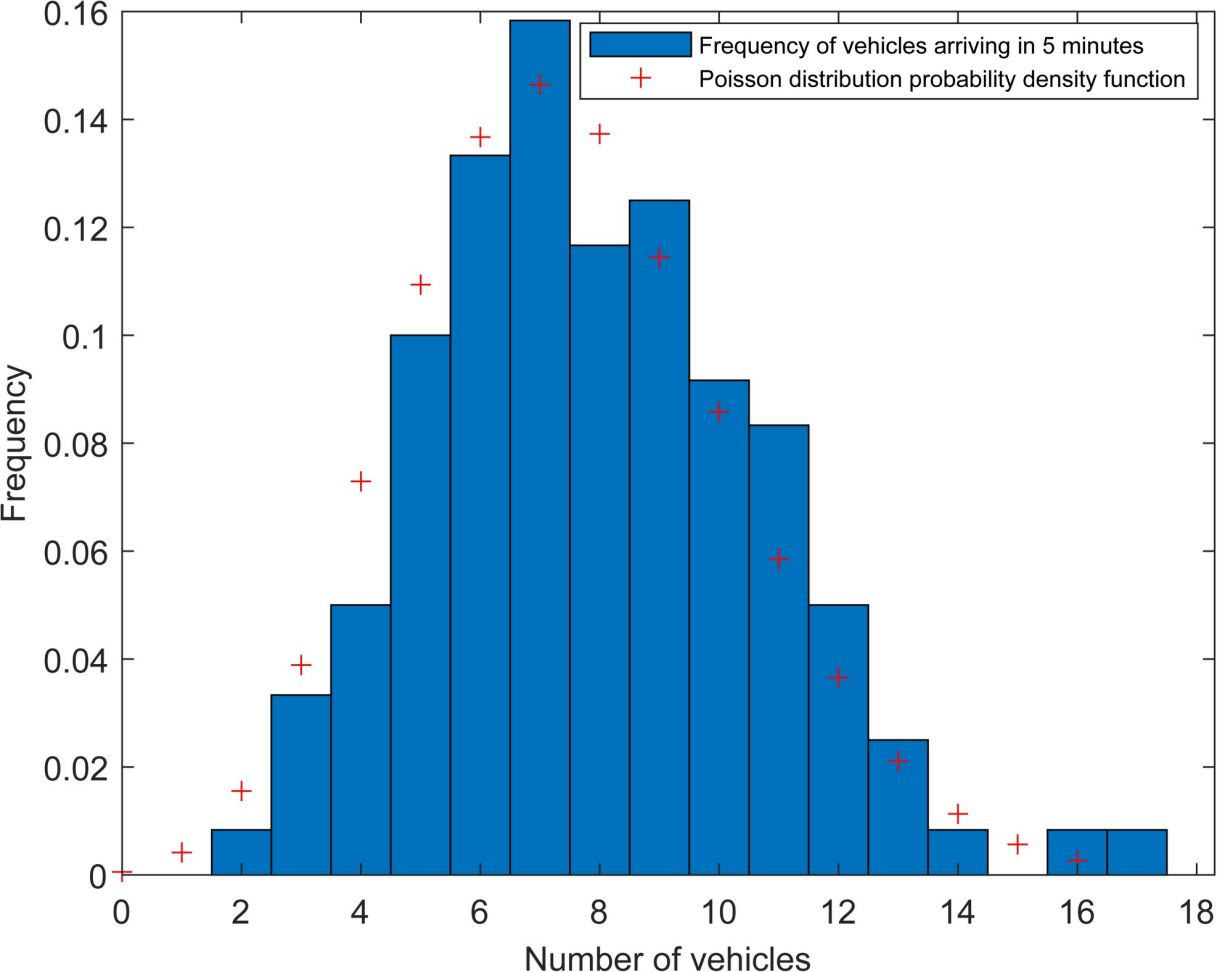
Vehicle arrival frequency distribution histogram.

The number of arrival vehicles during the survey period is counted in 5 minutes as a statistical unit. **Fig 1** shows the frequency distribution histogram of all samples at Monday. By comparing the probability density function with the Poisson distribution (the Poisson distribution parameter adopts the Maximum Likelihood Estimation Value, which is the mean of all the samples), it can be found that the morphologies of the samples and Poisson distribution are similar to some certain degree.

Arrival time of the consequent vehicles can be generally regarded as irrelevant to each other. The number of vehicle arrival during the non-intersecting time interval is statistically independent, which relates only to the interval duration and has nothing to do with the starting time. According to the characteristics of vehicle arrival, combing with the characteristics of Poisson process and the histogram of vehicle arrival frequency distribution (**Fig 1**), it is assumed that the vehicle arrival subjects to a Poisson distribution.

The K-S test method is adopted to examine whether the vehicle arrival obeys the Poisson distribution under 5% confidence level. The distribution parameters and test results are shown in **Table 1**, where *λ* is the distribution parameter denoting the average arrival frequency per unit time. It can be seen that all values of *p* are larger than 0.05, which means that the assume of Poisson distribution of vehicle arrival can be accepted.

**Table 1:** Vehicle arrival frequency distribution parameters and K-S test results.

Poisson distribution	Monday	Tuesday	Wednesday	Thursday	Friday
*λ*	7.93	7.11	6.65	8.59	7.53
K-S test value *p*	0.54	0.29	0.47	0.31	0.65

### Parking duration analysis

**Fig 2:**
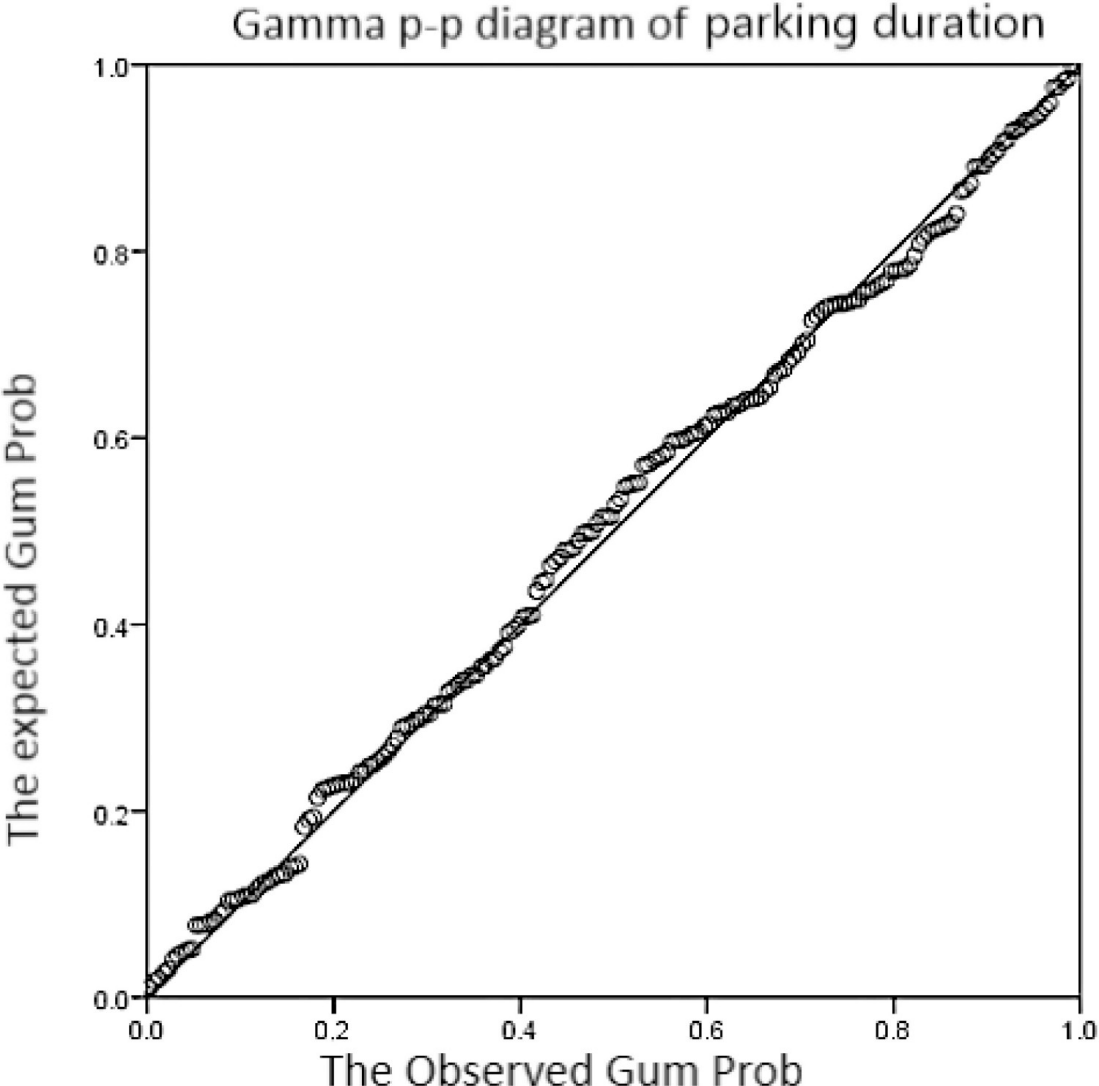
Gamma P-P diagram of the vehicle parking time on Monday and Tuesday in this commercial district.

Exponential Distribution, Gamma Distribution and other continuous distribution models are usually adopted to describe the parking duration. As seen from the P-P diagram of the Gamma Distribution in **Fig 2**, the distribution of the parking duration and the ideal gamma distribution have sufficiently slight difference. Then, gamma distribution can be considered to describe the distribution of parking duration, namely, Δ*t*~*G*(*α*, *β*), where *α* is the shape parameter, and *β* is the scale parameter.

Gamma distribution parameter is estimated using all the survey samples, and results are shown in **Table 2**. It can be known from the mean value that the average parking duration in commercial area is approximately 1.5 hours.

**Table 2:** Parameter distribution of the gamma distribution estimation of the all-day parking duration on weekdays.

Gamma distribution	Monday	Tuesday	Wednesday	Thursday	Friday
Shape parameter α	1.12	1.32	2.53	1.65	1.78
Scale parameter β	0.013	0.016	0.027	0.018	0.021
Mean value(minutes)	86.15	82.50	93.70	91.67	84.76

## Scenario description and parking sharing analysis

### Scenario description

Supposing that there is a closed parking plot in a residential area. An operation company purchases or rents the idle time of the parking spaces and sales the idle time to external users for extra income. A parking sharing platform should be introduced to collect the parking demand and parking supply. External users (the parking demand, not the owners of the parking spaces) and residents (the parking supply, owners of the parking spaces) should respectively submit the parking duration and the idle time to the sharing platform in advance according to their travel plans. To improve the utilization rate of the parking spaces, only an idle period more than 1 hour can be accepted by the platform for the residents.

When every external user submits his/her parking request, the platform will allocate the demand to the optimal place immediately according to the parking supply, which denotes that external users can obtain timely feedback from the platform. The real-time allocation mechanism implies that the allocation program has been determined simultaneously with parking demand generating.

Sometimes, because of some uncertain reasons, external users might not leave within the pre-submitted specified time (who are named as overtime users). Parking conflicts will emerge between the overtime user and the next user who has been allocated to the current parking space. To solve the parking conflicts, the operation company should reserve some parking spaces for the users who are affected by the overtime users. Overtime users will be additional charged to reduce the influence of overtime users as far as possible.

The parking user affected by overtime user will be assigned to the reserved parking spaces. However, because of the number limitation of reserved parking spaces and numerous overtime users, some users who have already been accepted in advance may be rejected and should be compensated economically by the platform. Additionally, the reserved shared parking spaces will be used only in cases of parking conflicts due to overtime users.

To obtain the maximum profits, the company must consider two primary factors, including the total number of parking spaces purchased or rented and reserved parking spaces, aiming to satisfy the parking demands as far as possible on the premise of maximizing the profits.

### Supply and demand analysis of shared parking spaces

**Fig 3:**
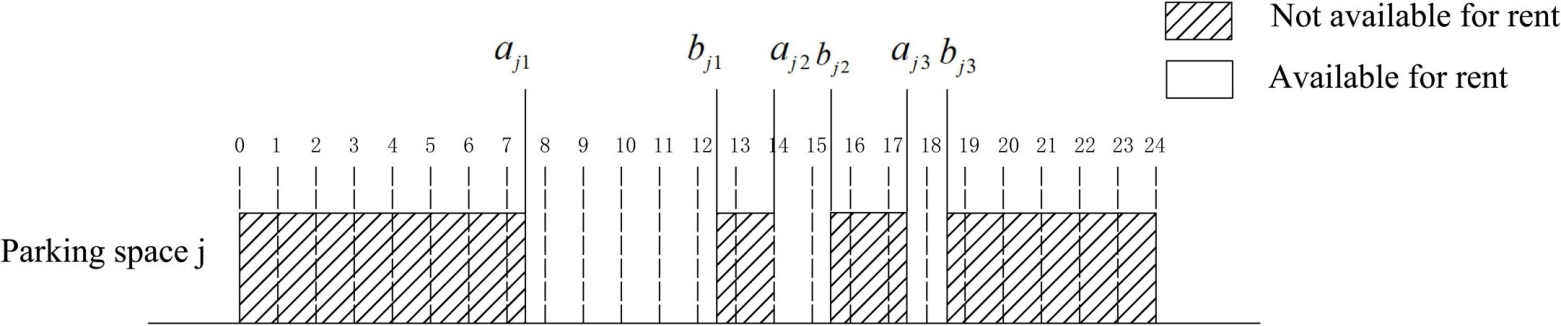
Schematic diagram of available free time for shared parking spaces.

In general, the available idle time of each parking space is different, and there can be one or more available idle time periods for one parking space. Assuming that there are *n* available parking spaces, the number of available time periods for parking space *j* is *q*_*j*_, and a_*jk*_,*b*_*jk*_ respectively represent the starting and ending time of the *k*^*th*^(*k*∈(1,⋯,*q*_*j*_)) available time period of parking space *j*. **Fig 3** is a schematic diagram of the idle time available for parking *j*, in which *q*_*j*_ = 3.

Here, *q* is the total number of all shared parking spaces’ available periods during the sharing time:

$$q=\sum_{j=1}^{n}q_{j}$$ (2)

*n* is the total number of shared parking spaces. At the initial moment, all the available periods of shared parking spaces can be expressed by a 2×*q* matrix *Q*:

$$Q=\left[\begin{array}{l} a_{11}\cdots a_{1k}\cdots a_{1q_{1}}\cdots a_{n1}\cdots a_{nq_{n}}\\ b_{11}\cdots b_{1k}\cdots b_{1q_{1}}\cdots b_{n1}\cdots b_{nq_{n}} \end{array}\right]$$ (3)

Assume *t*_*i*_ represents the starting time of parking demand *i* (that is the *i*^th^ external user), Δ*t*_*i*_ means the duration of parking demand *i*. The parking ending time is *t*_*i*_+Δ*t*_*i*_. *T*_*i*_~[*t*_*i*_,*t*_*i*_+Δ*t*_*i*_] denotes the parking duration of requirement *i*. Assuming that amount of parking requirements is *m* during a day, the parking requirements matrix *M* is defined as follows according to the order of parking demand submitting:

$$M=\left[\begin{array}{l} t_{1}\;\cdots t_{i}\;\cdots t_{m}\\ \left(t_{1}+\Delta t_{1})\cdots (t_{i}+\Delta t_{i})\cdots (t_{m}+\Delta t_{m}\right) \end{array}\right]$$ (4)

### Static allocation of shared parking requirements

Considering the convenience, the external users expect immediately feedback from the parking sharing platform after submitting their parking requirements. Therefore, a static allocation mechanism is conducted according to the submitting order; in other words, parking spaces are assigned for individual parking requirements. Before constructing the allocation model, some simplification has been proposed according to the parking characteristics and the limitations of factors considered in the problem. It is assumed that:

① Parking requirement users have no special preference for any parking space in the same parking garage.② The influence of different accessibility, safety and other factors of parking spaces is ignored. ③ All users must accept the system's allocation results.

Then the shared parking process and implementation are analyzed.

#### (1) Static allocation process of shared parking requirements

*1) Filtering the available idle time*. The parking demand period must be included within the idle periods of the shared parking spaces. Therefore, the idle periods that satisfy the time window constraint should be filtered first, namely:

$$(t_{i},t_{i}+\Delta t_{i})\in (a_{jk},b_{jk}),\;i\in m,j\in q$$ (5)

*2) Looking for the optimal idle time period*. Utilization maximization of all parking spaces has been taken as the basic principle of shared parking spaces allocation when there exist several available parking periods. Then, the allocation result should ensure the minimum gap g_*j*_ between the parking duration of the user and the idle time of parking spaces *j*.

$$\min(g_{j})=\max(g_{j1}=t_{i}-a_{jk},g_{j2}=b_{jk}-t_{i}-\Delta t_{i}),j=1,2,\cdots ,n;k=1,2,\cdots ,q_{j}$$ (6)

Then, the required parking demand *i* will be allocated to the *k*^th^ idle time period of parking *j* if the above condition is satisfied.

**Fig 4:**
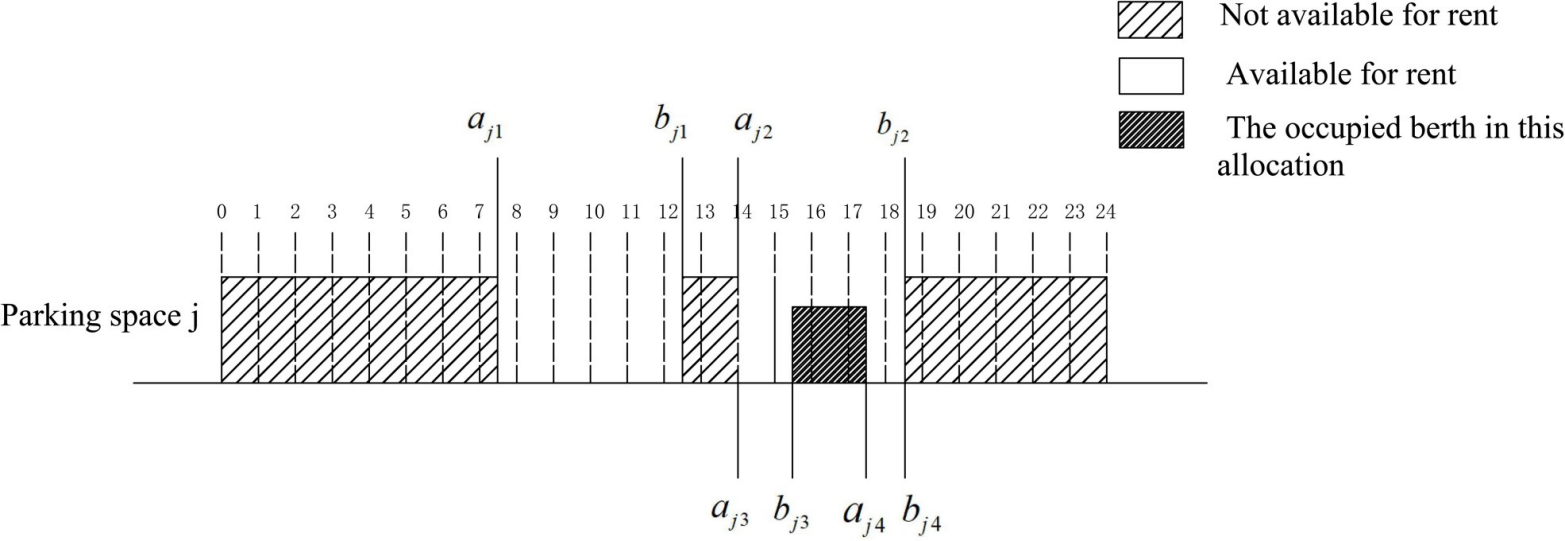
Update diagram of shared parking free time.

*3) Updating the idle time period*. When the *i*^th^ parking request is accepted and allocated to a certain parking space by the platform, the originally available idle time period will be divided into two periods, then it is necessary to update the matrix *Q* to allocate the next users. As shown in **Fig 4**, assuming that there are two available idle periods of parking space *j*, the parking demand *i* is successfully allocated to the second available period according to the principle of maximum utilization of parking spaces. Then, the second idle period is re-divided into two available periods including (*a*_*j*3_,*b*_*j*3_) and (*a*_*j*4_,*b*_*j*4_), and the matrix *Q* of parking space *j* is updated accordingly. Additionally, available parking periods of all parking spaces should be updated in matrix *Q* similarly after every parking demand is allocated to a certain parking space.

#### (2) If there are overtime users, the effected users will be re-allocated

**Fig 5:**
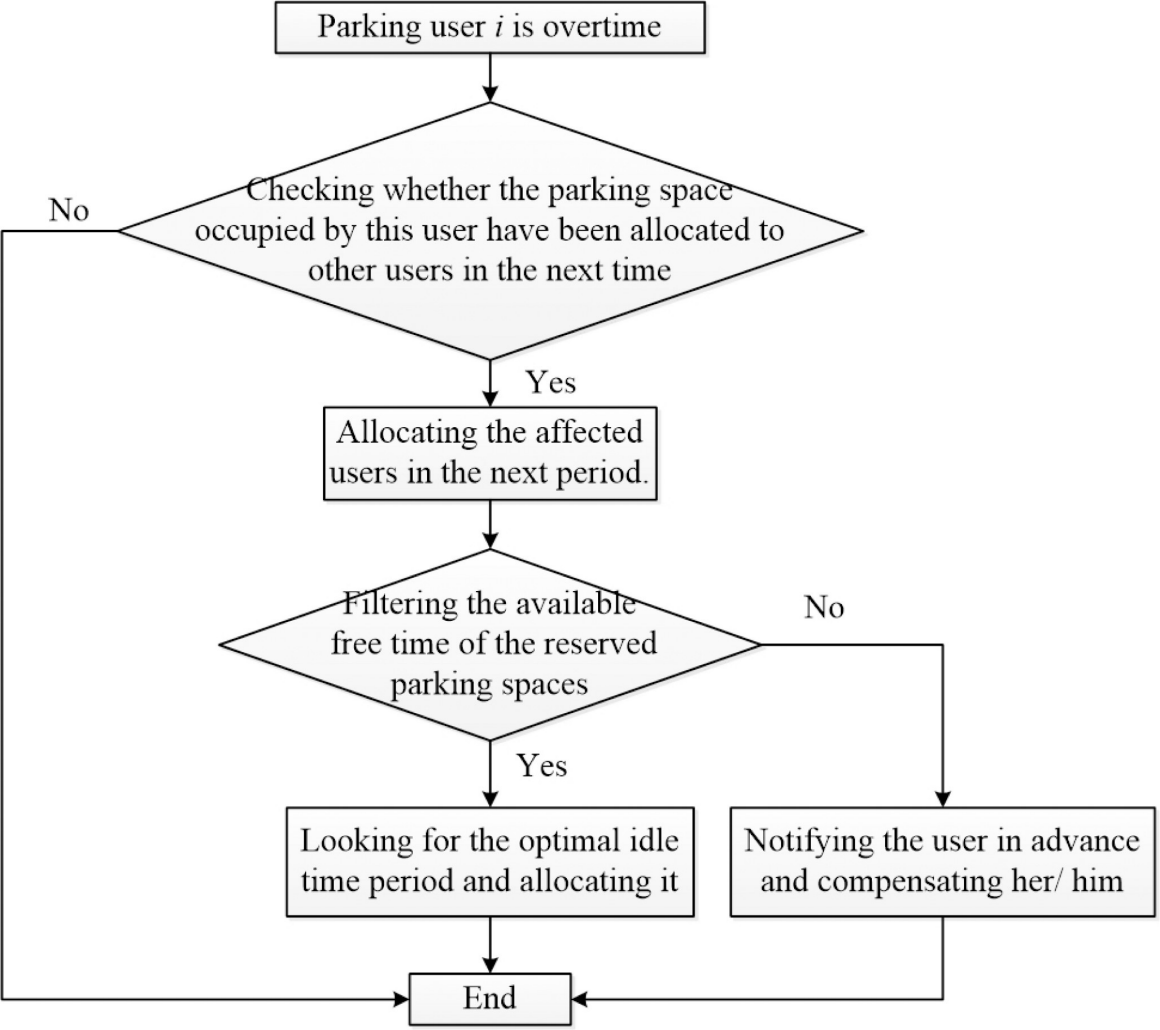
Shared parking allocation when there are overtime users.

If the parking spaces are sufficient for external users, the shared parking platform can still flexibly response to the parking conflict caused by overtime users and properly allocate the rest parking demand. Some parking spaces should be reserved to avoid the parking conflict caused by overtime users and the affected users will be re-allocated to the reserved parking spaces. The allocation principle is described in **Fig 5**.

## Sharing-parking allocation model establishment

### Definitions of variables and parameters

#### 1) Proportion of reserved parking spaces *ρ*

Reserved parking spaces are proposed to re-allocate the affected users by the overtime users. It is assumed that the proportion of reserved parking spaces among all of the shared parking spaces is *ρ*.

#### 2) Decision variable

$x_{ij_{k}}$ indicates whether the parking demand *i* is allocated to the *k*^th^ idle period of parking space *j*, where *i* = 1,2,⋯,*m*, *j* = 1,2,⋯,*n*, *k* = 1,2,⋯,*q*_*j*_.

xijk={1Parkingrequirementiisallocatedtothekthfreeperiodofparkingspacej0Parkingrequirementiisn'tallocatedtothekthfreeperiodofparkingspacej (7)

Due to the number limitation and restriction of the available time window of parking spaces, some external users will be rejected. *R*_*i*_ indicates whether the parking demand *i* is accepted by the platform; then,

$$R_{i}=\sum_{j=1}^{n(1-\rho )}\sum_{k=1}^{q_{j}}x_{ij_{{}_{k}}}\;(i=1,2,\cdots m)$$ (8)

*R*_*i*_ is a 0–1 variable. *R*_*i*_ = 1 means parking request *i* is accepted by the platform. Any parking request can only be allocated to one available period.

#### 3) Overtime length of external users

Here, $t_{i}^{leave}$ means the actual departure time of the *i*^*th*^ user. $t_{i}^{delay}$ is the overtime length of the *i*^*th*^ user, which can be expressed as follow.

$$t_{i}^{delay}=\max(t_{i}^{leave}-(t_{i}+\Delta t),0)\;(i=1,2,\cdots m)$$ (9)

If the *i*^*th*^ user leaves the parking space within the scheduled time, then $t_{i}^{delay}$ = 0.

Assuming that *D*_*i*_ is a 0–1 variable which indicates whether the parking demand *i* is an overtime user. We can include that, *D*_*i*_ = 1 if $t_{r}^{delay}\neq 0$, else *D*_*i*_ = 0. Some parking demand affected by overtime users may be rejected because of the number and periods constraint of the parking spaces. *Y*_*i*_ is proposed to denote the state of request *i*, and if request *i* is rejected by the platform because of overtime user, then *Y*_*i*_ = 1.

#### 4) Characteristic of parking periods

The parking time of external users is divided into three periods of peak, cross-peak and off-peak, and different parking charge rules are proposed based on the current parking policy. Cross-peak parking is charged according to low standard according to the principle of Xi 'an. Suppose *w*_*i*_ means the characteristic of parking time, if the parking time of request *i* belongs to the peak time, then *w*_*i*_ = 1, else *w*_*i*_ = 0.

### Objective function

The total benefit maximization has been considered as the objective of the allocation model, which is influenced by the acquisition cost of parking spaces, parking charge, overtime punishment fees and compensation fees. The acquisition cost means the charges that the operating company should pay to the owners for the parking spaces. External users have to pay parking charge according to the parking time. Overtime users should be extra-charged, which is called overtime punishment fee and higher than the normal parking fee. The operator should reimburse the users who are rejected by the platform because of overtime users, that is the compensation fee. The fixed investment cost and other variable costs of the parking spaces are ignored in the model.

Additionally, we assume that the owners of the parking spaces will not return early and strictly observe the submitted plan. Then the overtime users only indicate the external users and parking conflict only occur among the external users.

Then, binary integer linear programming can be used to express the revenue function of the shared parking system:

$$\mathrm{maxZ}=P_{a}\sum_{i=1}^{m}R_{i}\Delta t_{i}+\Delta P_{1}\sum_{i=1}^{m}R_{i}w_{i}\Delta t+\Delta P_{2}\sum_{i=1}^{m}R_{i}t_{i}{}^{delay}-P_{b}\sum_{j=1}^{n}\sum_{k=1}^{q_{j}}\left(b_{jk}-a_{jk}\right)-P_{c}\sum_{i=1}^{m}Y_{i}$$ (10)

where $R_{i}=\sum_{j=1}^{n(1-\rho )}\sum_{k=1}^{q_{j}}x_{ij_{k}}$, $t_{i}^{delay}=\max(t_{i}^{leave}-(t_{i}+\Delta t),0)$,

*P*_*a*_——Basic parking fee during off-peak period, yuan/(billing unit),

Δ*P*_1_——Floating parking fee during peak period, yuan/(billing unit),

Δ*P*_2_——Overtime punishment fee for overtime users, yuan/(billing unit),

*P*_*b*_——Acquisition cost for a parking space, yuan/(billing unit),

*P*_*c*_——Compensation fee for users who are rejected because of overtime users, yuan/vehicle.

### Constraint conditions

#### 1) Optimal allocation program constraint

The constraint of the time window should be satisfied first. Then, external user should be allocated to the parking space with minimum idle period gap. If there are several optimal shared parking spaces, then one of them should be randomly selected. There are the following:

$$x_{ij_{k}}=\left\{ \begin{array}{l} 1\;(t_{i},t_{i}+\Delta t_{i})\in (a_{jk},b_{jk})\cup \min(g_{j})\\ 0\;(t_{i},t_{i}+\Delta t_{i})\notin (a_{jk},b_{jk}) \end{array}\right.$$ (11)

#### 2) Number constraint of shared parking spaces

The occupancy rate of the parking is relatively high in the peak parking periods, then the total number of accepted external users during the peak period should not exceed the total number of parking spaces available.

$$\sum_{i=1}^{m}w_{i}R_{i}\leq n(1-\rho )$$ (12)

#### 3) Number constraints of the reserved parking spaces

Similarly, number of reserved parking spaces should be more than the number of overtime users.

$$n\rho \geq \sum_{i=1}^{m}w_{i}D_{i}$$ (13)

### Shared parking allocation model

According to the above analysis, the shared parking allocation model aiming at benefit maximization can be expressed as:

$$\mathrm{maxZ}=P_{a}\sum_{i=1}^{m}R_{i}\Delta t_{i}+\Delta P_{1}\sum_{i=1}^{m}R_{i}w_{i}\Delta t+\Delta P_{2}\sum_{i=1}^{m}R_{i}t_{i}^{delay}-P_{b}\sum_{j=1}^{n}\sum_{k=1}^{q_{j}}\left(b_{jk}-a_{jk}\right)-P_{c}\sum_{i=1}^{m}Y_{i}$$ (14)

where $R_{i}=\sum_{j=1}^{n(1-\rho )}\sum_{k=1}^{q_{j}}x_{ij_{k}}$, $t_{i}^{delay}=\max(t_{i}^{leave}-(t_{i}+\Delta t),0)$

$$s.t\;\left\{ \begin{array}{l} x_{ij_{k}}=\left\{ \begin{array}{l} 1\;(t_{i},t_{i}+\Delta t_{i})\in (a_{jk},b_{jk})\cup \min(g_{j})\\ 0\;(t_{i},t_{i}+\Delta t_{i})\notin (a_{jk},b_{jk}) \end{array}\right.\\ n\rho \geq \sum_{i=1}^{m}w_{i}R_{i}\\ \sum_{i=1}^{m}w_{i}D_{i}\leq n(1-\rho )\\ w_{i},D_{i},R_{i},Y_{i}\in \left\{0,1\right\} \end{array}\right.$$

### Simulation analysis

Some assumptions are proposed first according to the actual parking behavior and the analysis above:

1) Taking the residential area as an example, the idle time on workdays is mostly concentrating between 9:00 a.m. and 17:00 p.m. Then, the initial available period of all shared parking lasts from 9:00 a.m. to 17:00 p.m.. The sharing period only includes the morning peak hour, which lasts from 9:00 a.m. to 10:00 a.m..

2) The number of arriving vehicles is subject to Poisson distribution and the distribution parameter λ is randomly valued among [7,9] according to Table 1.

3) The parking duration of external users is subject to gamma distribution, and the average parking duration is 1.5 hours.

4) Billing unit of parking charge is 30 minutes, which is in line with the current parking charge standard in Xi 'an.

Other basic parameters are shown in Table 3 and the values of parking charge refers to the parking charge standard in Xi’an.

**Table 3:** Parameters and values of basic scenario.

Parameters	Explanation
*n =* 50	Total number of shared parking spaces
*q* = 50	Initial number of available idle time periods
*P*_*b*_ = 1yuan/(30min)	Purchase cost of a shared parking space
*P*_*a*_ = 2yuan/(30 min)	Basic parking fee during off-peak hours
Δ*P*_1_ = 1yuan/(30min)	Floating fee during peak hours
Δ*P*_2_ = 2yuan/(30min)	Floating fee for external uses
*P*_*c*_ = 10 yuan/vehicle	Compensation fee for the affected external users

#### (1) Proportion of reserved parking

**Fig 6:**
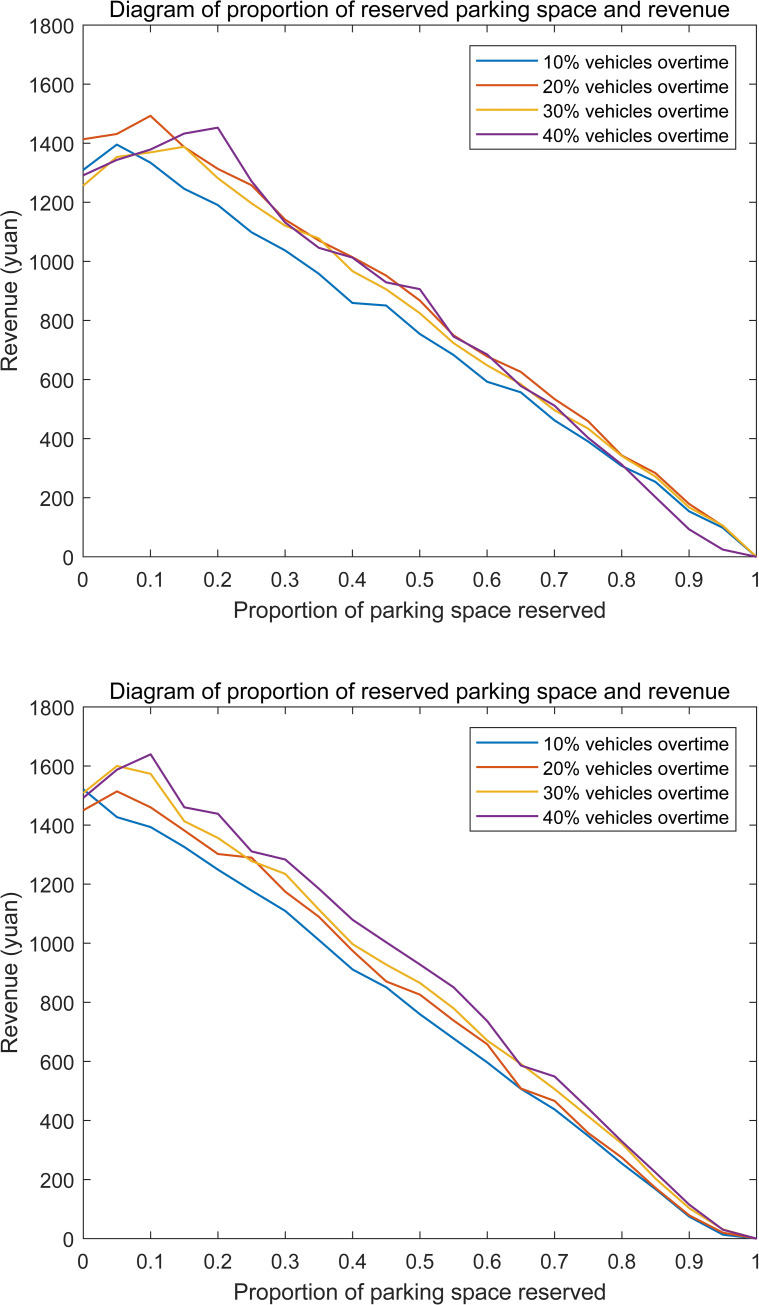
(a) Relationship between the proportion of reserved parking spaces and the revenue. (b) Relationship between the proportion of reserved parking spaces and the revenue.

Based on the assuming that the external parking demand is constant and larger than the shared parking supply, the optimal proportion of reserved parking can be determined. The average overtime length of the overtime users is considered as the constant because of a lack of actual data on shared parking. The two conditions of $t_{i}^{delay}$ = 1h and $t_{i}^{delay}$ = 1.5h in the basic scenario are analyzed. Relationship between the proportion of reserved parking and the total revenue is shown in **Fig 6** and the corresponding maximum revenues under different proportion of overtime users are shown in **Table 4**.

**Table 4:** Optimal proportion of reserved parking and maximum revenue under different proportion of overtime users when $t_{i}^{delay}$ = 1h.

Proportion of overtime users	10%	20%	30%	40%
Optimal proportion of reserved parking space	0.05	0.1	0.15	0.2
maximum revenue(yuan)	1395	1493	1388	1453

Obviously, more parking spaces should be reserved in advance when number of overtime users increase. However, there is little difference among the maximum revenues corresponding to different proportions of overtime users. That is because the compensation for rejected users can be basically offset by the extra-charge for overtime users. Additionally, when number of overtime users is fixed, revenue is gradually declined with the proportion of reserved parking spaces increasing, because the utilization of reserved parking spaces decreases. We can conclude that, optimal proportion of reserved shared parking approximately equals to half of the proportion of overtime parking users.

**Fig 7:**
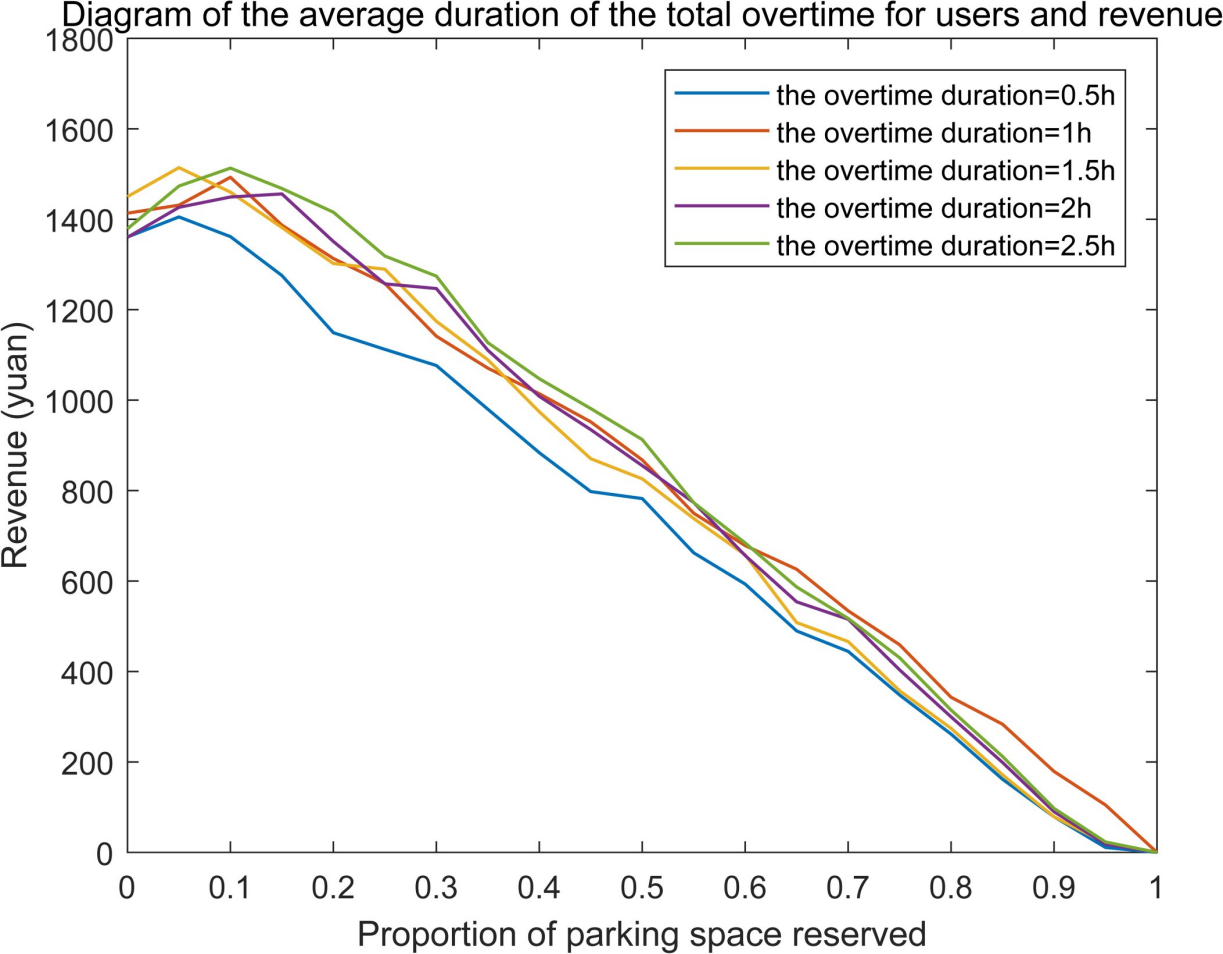
Relationship between the overtime duration and revenue when the proportion of overtime users is 20%.

Different values of overtime duration are deeply analyzed to reveal the influence of overtime parking behavior on the total revenue under condition of fixed proportion of overtime users, which is shown as **Fig 7**.

We can find that, there always exists an optimal proportion of reserved parking spaces and maximum revenue no matter the overtime duration. However, the optimal proportion of reserved parking spaces corresponding to the maximum revenue is not always increased with the overtime duration increase. We can infer that, rejected users will increase with the increasing of overtime duration and the manager should compensate more for the rejected users. However, the extra-charge parking fee of overtime users increases at the same time. The benefit of extra-charge parking fee may offset the loss of rejected users to some extent. Therefore, the needed reserved parking spaces will not increase necessarily. Similarly, there is little difference among the maximum revenues corresponding to different proportions of overtime users.

#### (2) Number of parking request

**Fig 8:**
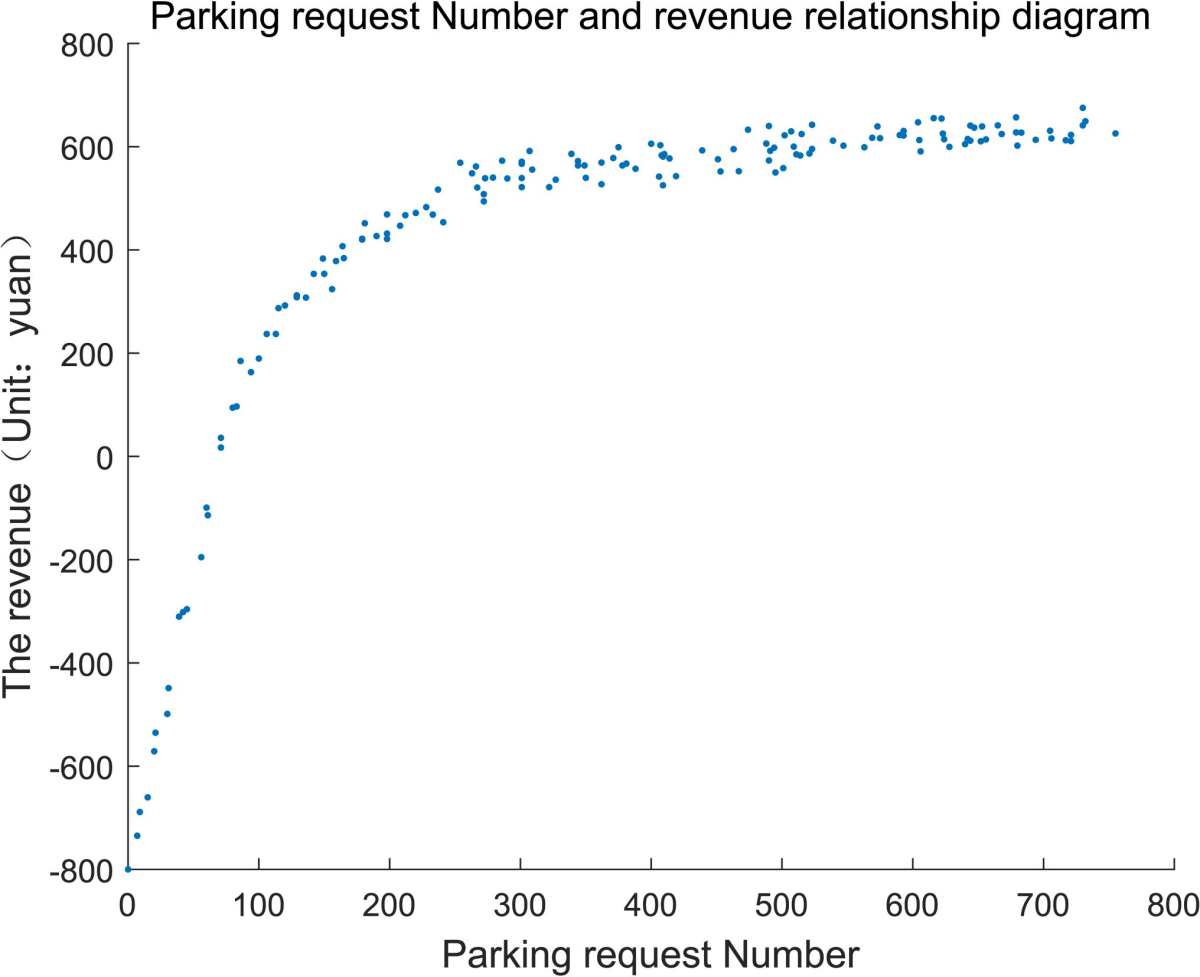
Parking request number and revenue relationship diagram.

**Fig 9:**
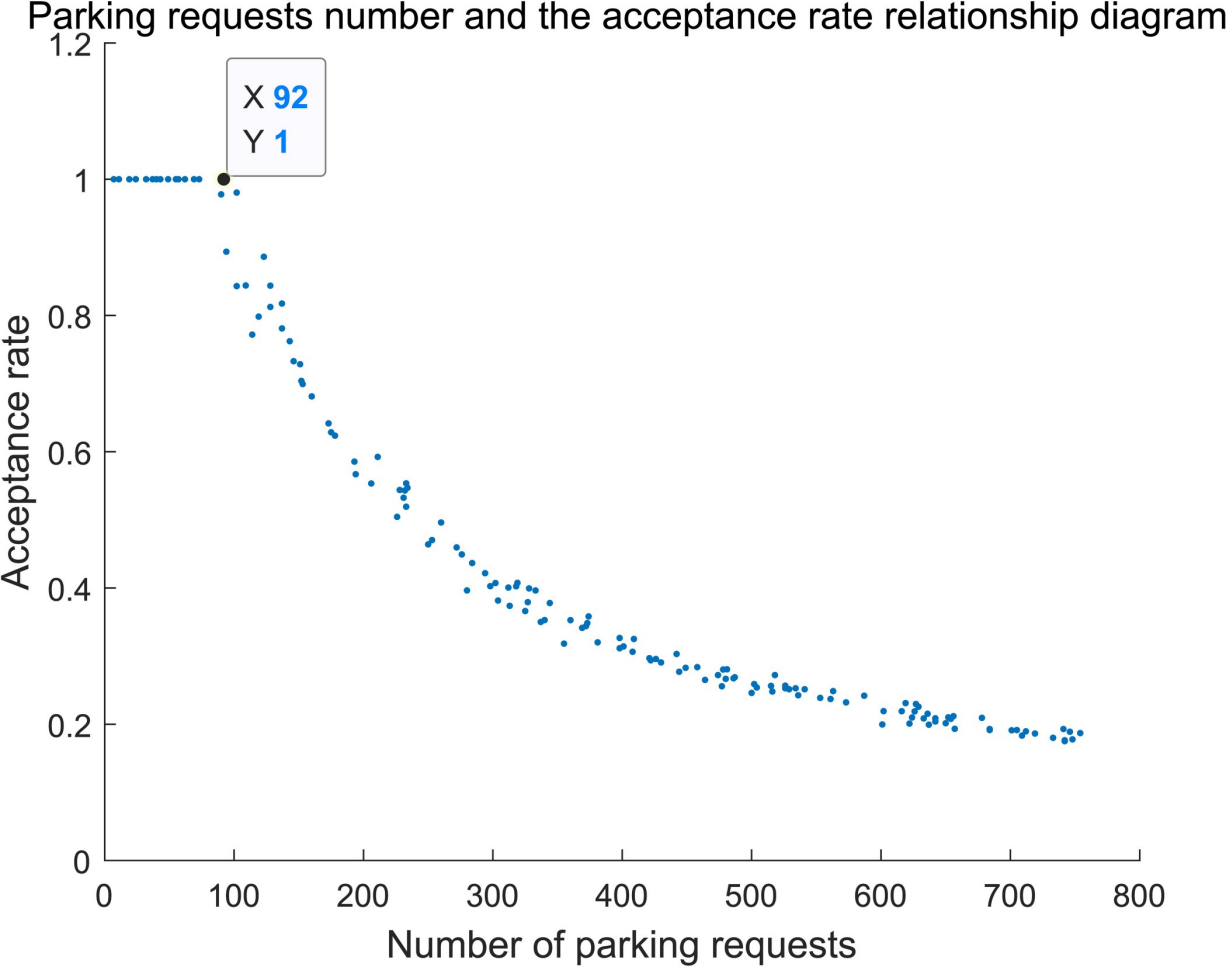
Parking request number and the acceptance rate relationship diagram.

Based on the analysis of Table 4, it is assumed that the proportion of overtime users is 20% and the optimal proportion of reserved parking is *ρ* = 10%. The relationships among the revenue, the acceptance probability of external users and parking request are shown in **Figs 8** and **9**, respectively.

The variation tendency shown as **Figs 8** and **9** indicates that both the total revenue and acceptance probability can be divided into three stages. 1) The first stage: total revenue increases linearly as the number of parking requests increases when parking request is little. The initial negative revenue derives from the large parking acquisition cost and little parking revenue. At the stage, increase rate of revenue is 6 yuan/vehicle, which is approximately equal to the product of average parking duration and unit parking fee. The acceptance proportion is 100%; in other words, all of the parking requests can be accepted. 2)The second stage: the total revenue shows nonlinear growth when the parking demand exceeds 92 vehicles, and the acceptance rate gradually decreases. Because the parking request is selectively rejected, the revenue will increase continually. 3)The third stage: the total revenue remains basically stable and slightly fluctuates when parking request exceeds 351, acceptance rate continues to decline. The accepted parking demand is saturated because of the number constraint of shared parking spaces, then the total revenue reaches the maximum value and keeps floating within a small range.

The process can be shown in **Table 5**:

**Table 5:** Relationship between the number of parking requests and the revenue.

Stage	The first stage	The second stage	The third stage
Parking demand (vehicle)	0~92	92~351	351~800
Total revenue	Linear growth	Nonlinear growth	Keep stable and slightly fluctuate
Acceptance rate	= 1	Decline rapidly	Continue to decline
Ratio of parking demand to number of shared parking	0~1.84	1.84~7.02	>7.02

It can be found that, there exists the maximum revenue when the parking demand increases gradually. Under the given scenario above, 351 parking requests can lead to the maximum revenue, and the corresponding acceptance rate of parking users is about 35%.

**Fig 10:**
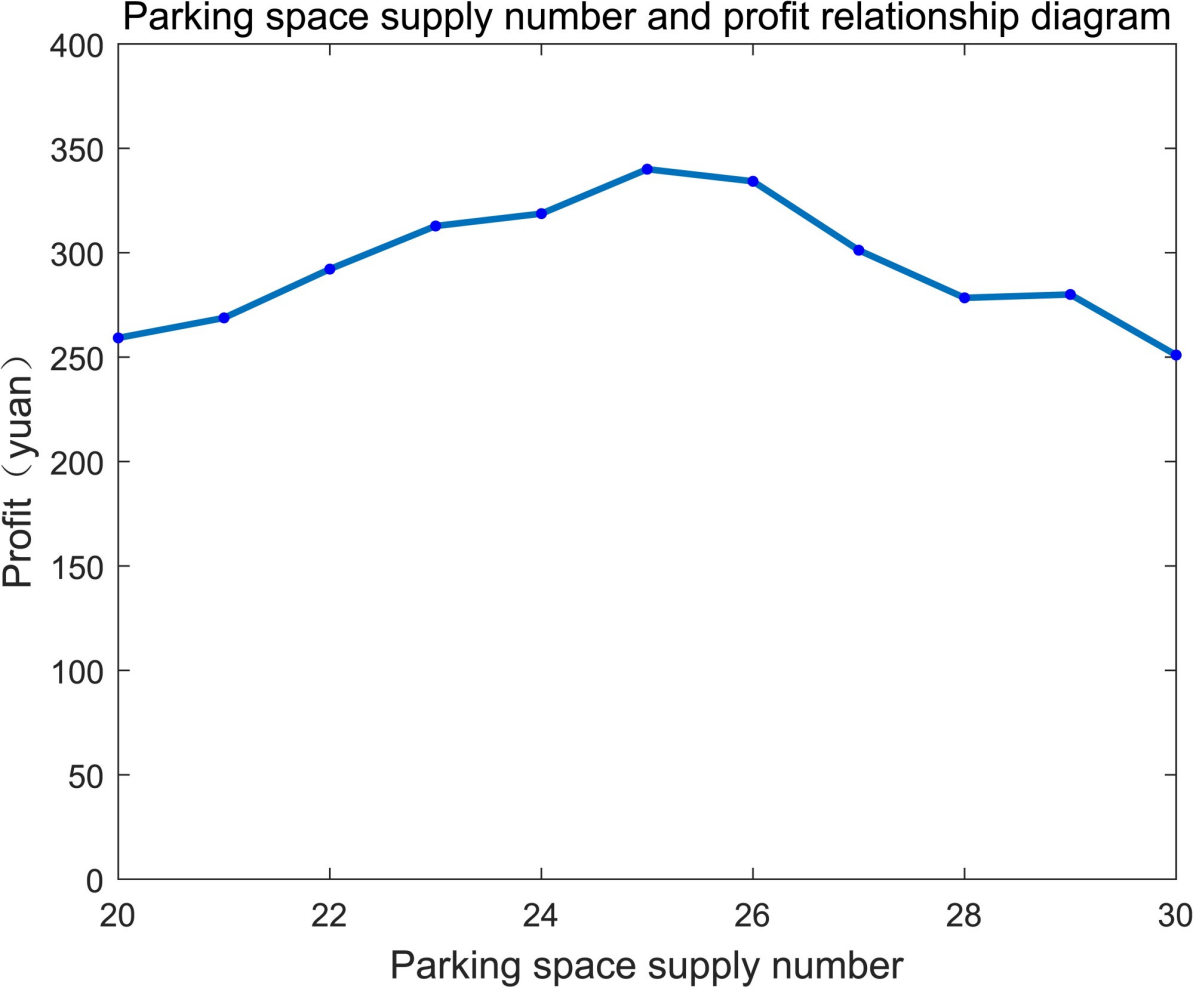
Relationship between shared parking space supply and profit.

In order to reduce the purchase cost and improve the utilization of parking spaces, operators should determine the optimal number of shared parking. The parking demand of the surrounding area is about 200 vehicles/day according to the investigation data, the revenue under different numbers of shared parking is shown in **Fig 10**.

We can include that the sharing revenue can be maximized when the number of shared parking spaces is equal to 25(that is *n* = 25). When shared parking spaces are less than 25, revenue increases with the shared parking spaces, because less parking spaces cannot satisfy all of the demand and the acceptance is less than 1. The revenue declines gradually when the parking spaces exceed 25, because the parking supply outnumbers the demand and the utilization of the parking spaces will descend.

#### (3) Shared parking utilization analysis

The shared parking utilization rate *η* can be expressed as:

$$\eta =\frac{\sum_{i=1}^{m}R_{i}\Delta t_{i}}{\sum_{j=1}^{n}\sum_{k=1}^{q_{j}}\left(b_{jq_{j}}-a_{jq_{j}}\right)}$$ (15)

**Fig 11:**
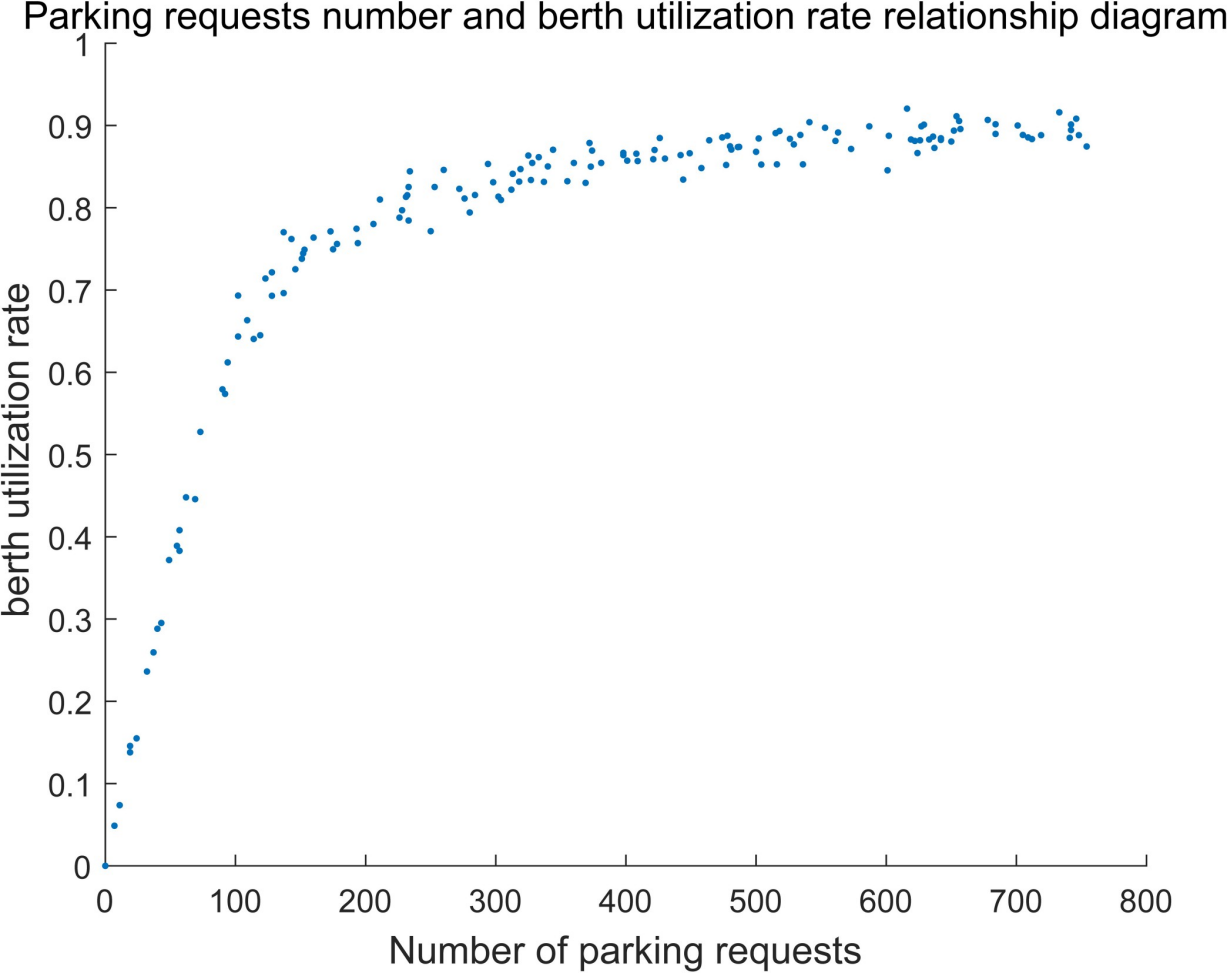
Relationship between parking requests and shared parking utilization rate.

The relationship between shared parking utilization and the parking requests is shown in **Fig 11.**

As shown in **Fig 11**, the variation of shared parking utilization can also be divided into three stages. In the first stage, parking spaces are enough for the parking demand, and parking utilization increases linearly. In the second stage, parking utilization nonlinearly increases because some requests are rejected when the parking requests continue to raise. In the third stage, utilization reaches the maximum and almost stays unchanged. That is because all of the parking spaces can be utilized when demand outnumbers the shared parking spaces. Additionally, the maximum utilization of shared parking is approximately equal to 90%, which indicates that not all of the parking spaces can be fully utilized. Because some parking demand cannot be completely matched to a certain parking space, then not all the parking spaces are fully utilized even if the parking requests exceeds the supply.

#### (4) Relationship among the revenue, parking duration and utilization of shared parking spaces

Parking duration is the critical factor influencing the revenue and utilization of the shared parking, then the effects of different parking duration are deeply analyzed. Table 6 shows the relationship among the maximum revenue, the corresponding parking demand and the maximum utilization rate of the shared parking spaces under different parking durations.

**Table 6:** Parking revenue and utilization under different parking durations.

Average parking duration	1 h	1.5 h	2 h	2.5 h	3 h
Maximum Revenue (yuan)	693	681	676	673	660
Parking demand corresponding to the maximum revenue (vehicle/day)	512	440	415	396	317
Maximum utilization of shared parking spaces	0.9	0.89	0.88	0.88	0.88

As seen from **Table 6**, the maximum utilization and revenue slightly decline with the increase of parking duration. In other words, the maximum parking revenue has no obvious difference corresponding to different parking durations, as same as the utilization. However, parking demand corresponding to the maximum revenue declines greatly as the increase of average parking duration, which denotes that even small parking demand can bring approximately maximum benefits through accepting vehicles with longer parking duration preferentially. Therefore, the users with longer parking duration have priority to be accepted by the platform when there exists a large amount of parking demand. The selectively acceptance strategy can obtain approximately maximum revenue and utilization simultaneously under less acceptance of external users, which can reduce the shared parking garage’s turnover and minimize the influence of external parking on the residents. Both the operator and owners of the parking spaces can obtain the maximum economic and social benefit.

## Conclusions

Shared parking has been introduced to make full use of parking resources. Residential areas adjacent to mixed commercial land has been taken as an example of parking sharing in the article. A benefit maximum model considering overtime parking has been established based on the allocation principle of maximum utilization of shared parking spaces. The relationships among the parking demand, parking duration, number of shared parking spaces and total parking revenue are analyzed deeply. The conclusions are summarized as follows:

(1) The optimal proportion of reserved shared parking similarly equals to half of the proportion of overtime parking users, and the corresponding total parking revenue will increase as the number of overtime users increase.

(2) The tendency of shared parking revenue and the acceptance rate with increasing parking demand shows some periodic changes. Parking revenue increases linearly when the parking demand increases within a certain threshold, and the acceptance rate of the parking demand equals to 1. When the parking demand exceeds the threshold, parking revenue nonlinearly increases and the acceptance rate of the parking demand decreases with the increase of parking demand. When the parking demand exceeds another threshold and continues to increase, parking revenue will stay stable and fluctuate slightly, and the acceptance rate of the parking demand continues to decrease. The changing tendency denotes that there exists an appropriate shared parking demand corresponding to the maximum revenue.

(3) There exists an optimal number of shared parking spaces ensuring that the operator can obtain the maximum revenue.

(4) Utilization rate of shared parking shows a periodic change when the parking demand increases, including initial linear increase, mid-term nonlinear increase and final stable state, and the saturation utilization rate approximately equals to 90%.

(5) The maximum utilization and revenue of shared parking remain almost unchanged as the average parking duration increases, but the number of external parking demand that can be accepted corresponding to the maximum revenue gradually decrease. External demands with longer parking duration should be accepted preferentially, which can obtain the similarly maximum profit and utilization of parking spaces, but reduce the impact of external users on residents at the same time.

The managers can determine the optimal number of purchased shared parking and the proportion of reserved shared parking based on the maximum revenue according the optimization model. Then, operator can decide the optimal operation scheme to guarantee the greatest benefit both for the manager and residents.

However, parking characteristics of parking duration and arrival time are described separately and the complexly intricate relations have not been considered. Simulation method is used to describe the optimal model and all parameters are given by hypothesis, which may not reveal the real shared parking behavior. In addition, dynamic adjustment should be considered in the improved model. In future research, real shared parking scenarios and dynamic allocation scheme will be paid more attention on.

## Supporting information

S1 Data The supplement material includes the arrival time, leaving time and vehicle state (arrival or leaving). All vehicles have been matched through the license plate number, and the license plate number is anonymous considering personal privacy.
(XLSX)
