# Correction: Research on parking sharing strategies considering user overtime parking

**DOI:** 10.1371/journal.pone.0251807

**Published:** 2021-05-11

**Authors:** Xin Huang, Xueqin Long, Jianjun Wang, Lan He

After publication of the article, the authors noticed an error in formula 14. Incorrect constraints were transcribed into the formula when the assignment rules were transplanted into the assignment model. The errors affect the assignment regulation.

In the Shared parking allocation model subsection of Sharing-parking allocation model establishment section, there are errors in formula 14. The constraints of formula 14 were derived from formulas 10, 11 and 12. The second constraint is the same as formula 11, and the third constraint is the same as formula 12. Due to a transcription error, the second and third constraints are incorrect. Only these two constraint formulas are incorrect. The input results in the article are correct. Please view the complete, correct formula here:

$$\mathrm{maxZ}=P_{a}\sum_{i=1}^{m}R_{i}\Delta t_{i}+\Delta P_{1}\sum_{i=1}^{m}R_{i}w_{i}\Delta t+\Delta P_{2}\sum_{i=1}^{m}R_{i}{t_{i}}^{delay}-P_{b}\sum_{j=1}^{n}\sum_{k=1}^{q_{j}}\left(b_{jk}-a_{jk}\right)-P_{c}\sum_{i=1}^{m}Y_{i}$$ (14)

where $R_{i}=\sum_{j=1}^{n(1-\rho )}\sum_{k=1}^{q_{j}}x_{ij_{k}},\;{t_{i}}^{delay}=\max({t_{i}}^{leave}-(t_{i}+\Delta t),0)$

$$s.t\;\left\{ \begin{array}{l} x_{ij_{k}}=\{\begin{array}{l} 1\;(t_{i},t_{i}+\Delta t_{i})\in (a_{jk},b_{jk})\cup \min\;(g_{j})\\ 0\;(t_{i},t_{i}+\Delta t_{i})\notin (a_{jk},b_{jk}) \end{array}\\ n(1-\rho )\geq \sum_{i=1}^{m}w_{i}R_{i}\\ \sum_{i=1}^{m}w_{i}D_{i}\leq n\rho \\ w_{i},D_{i},R_{i},Y_{i}\in \{0,1\} \end{array}\right.$$
